# Pregnancies with an outcome of fetal death present higher risk of delays in obstetric care: A case-control study

**DOI:** 10.1371/journal.pone.0216037

**Published:** 2019-04-29

**Authors:** Marley Carvalho Feitosa Martins, Francisco Edson de Lucena Feitosa, Antonio Brazil Viana Júnior, Luciano Lima Correia, Flávio Lúcio Pontes Ibiapina, Rodolfo de Carvalho Pacagnella, Francisco Herlânio Costa Carvalho

**Affiliations:** 1 Department of Community Health, Federal University of Ceará, Fortaleza, Ceará, Brazil; 2 Department of Maternal and Child Health, Federal University of Ceará, Fortaleza, Ceará, Brazil; 3 Center for Health Sciences, University of Fortaleza, Fortaleza, Ceará, Brazil; 4 Department of Tocoginecology, State University of Campinas, Campinas, São Paulo, Brazil; Aga Khan University, KENYA

## Abstract

The objective of this study was identify the association between delays in the care provided to pregnant women and the fetal death outcome, in a tertiary reference maternity hospital in the Northeastern Brazil. A case-control study, with 72 cases of fetal death and 144 controls (live births) in women admitted to the Obstetrics Service of the Assis Chateaubriand Teaching Maternity Hospital, in Fortaleza, Ceará. Controls were matched (2:1) by the approximate gestational age of the case. The groups were compared using the three delays model of obstetric care. The Pearson's Chi-square test and the Fisher's exact test were used to compare the groups. P <0.05 was considered statistically significant. The Group with fetal death had a smaller number of prenatal consultations (> 6 consultations: 27.8% in cases, 40.3% in controls, p = 0.003), less risk classification of pregnancy (41.7% vs 55.9%, p = 0.048), less guidance about the health facility for delivery (44.5% vs 64%, p = 0.009), lower frequency of cesarean sections (25.4% vs 65.7%) and higher frequency of hemorrhagic syndromes (33.3% vs 19.4%, p = 0.024) and syphilis (15.3% vs 4.2%, p = 0·004). Variables that persisted significantly associated with fetal death in the logistic regression were: Refusal of assistance (OR = 4.07, IC 95%: 1.08–15.3), Absence or inadequacy of prenatal care (OR = 2.69, IC 95%: 1.07–6.75), Delay in diagnosis (OR = 10.3, IC 95%: 2.58–41.4) and Inadequate patient conduct (OR = 4.88; IC 95%: 1.43–16.6). Despite of having a higher frequency of obstetric complications, gestations with fetal death are more prone to delays in obstetric care.

## Introduction

According to international estimates, Brazil is in an intermediate range of fetal mortality rate: five to 14.9 deaths per thousand births. However, the low quality of the information and the under-registration of fetal deaths in the official systems compromise the real dimensioning of the problem. In many health services, there are still no routines for the analysis of the occurrence of fetal death, as well as lack of specific investments for its reduction. This reality results in the difficulty of knowing the factors that cause the death of these fetuses, making it difficult to develop intervention measures [1].

Data from the Brazilian Northeastern region, in addition to identifying deficiencies in prenatal care and delivery, showed that 25% of pregnant women had to seek more than one maternity at the time of delivery, a problem that poses a potential risk to maternal and fetal life. About 95% of all fetal deaths occurred in this region were codified in Chapter XVI of the International Classification of Diseases (ICD-10) as "Some conditions originating in the perinatal period", which are strongly influenced by the access and quality of care offered to women during pregnancy and childbirth [1].

In case of obstetric emergency, each moment of delay in the search for appropriated care, increases the risk of maternal and fetal death [2]. The assistance assessment approach, [3] known as "Three Delays", applied to stillbirths, was utilized in the current study [4]. This model links mortality to a series of delays in obstetric care, which prevent women from having access to skilled and effective care in a timely manner [5].

In Brazil, generally, the "Three Delays" model has been utilized for analysis of maternal mortality data and cases with severe maternal outcomes (Near miss) [5]. In the international literature, three studies of perinatal deaths were carried out using the analysis of three delays: one carried out in Rwanda [6], other in Tanzania [7], and a meta-analysis of delays in obtaining effective care during labor and at birth [2].

The objective of this study was to identify the association between delays in the care provided to pregnant women and the fetal death outcome, in a tertiary reference maternity hospital in the Northeastern Brazil.

## Materials and methods

A case-control study was carried out from the prospective surveillance of cases of fetal death and live births in women admitted to the Obstetrics Service of the Assis Chateaubriand Teaching Maternity Hospital of the—Federal University of Ceará.

This tertiary reference hospital provides specialized care for high-risk pregnant women living in the state of Ceará, Northeastern Brazil, performing about 5,000 births per year. It has Fetal Medicine Services and a Maternal and Neonatal Intensive Care Unit, being considered by the Ministry of Health as Reference Center for Good Obstetric and Neonatal Care Practices.

The study sample size comprised 72 cases and 144 controls. The cases of fetal death occurred from January to November, 2017, were recruited for the group of cases, while the controls were select among the live births occurred in the same period, paired by gestational age, following an 1:2 ratio. The inclusion criteria used for the cases were: diagnosis of fetal death confirmed by ultrasound (before or after hospital admission) occurring from the 22^nd^ full week of gestation, with birth weight equal to or greater than 500g. For the controls, the criterion was being born alive, with maternal hospitalization, in the same period of the cases. Women who had any physical and/or mental incapacity, that made the interview unfeasible, and women under 18 years of age who were not accompanied by their parents, were excluded. The criterion of eligibility common to the cases and controls was that the births were performed in the maternity hospital.

Cases and controls were identified from the daily birth register of maternity hospital neonatology unit. Once eligibility was ascertained, the women were invited to participate in the study and those who agreed to participate (or their legal guardian) signed an Informed Consent Term.

The variables selected for the study were collected through interview after birth, using a standardized questionnaire that included sociodemographic and obstetric data, and factors related to delays in the provision of obstetric care. The history of the fetal loss was also collected from the beginning of the problem until the arrival at the maternity, for the group of cases. The criteria for the analysis of the "Three Delays" were defined based on the criteria standardized by World Health Organization (WHO) [8]. These involved questions related to the delay in recognizing the need for care (Delay in seeking care, not knowing the signs of risk, refusal of care), delay in access to care (absence or inadequacy of prenatal care, difficulty with transportation, geographical difficulty) and delay in receiving quality care in the unit (delay in diagnosis, difficulty in communication between hospital and regulatory center, lack of trained personnel, delay in the referral / transfer of the case, delay in starting treatment, inadequate conduct with the patient).

Information on third phase delays was collected from medical records and other registers such as Death Certificate and pregnancy card. To define delays in medical conduct ("delay in diagnosis" and "delay in initiating treatment") two specialists in high-risk gestation monitoring at the maternity had to agree, after analyzing the medical records without any prior knowledge of the final outcome (as to which group was involved: cases or controls).

In the analysis cases and controls were compared using the Pearson Chi-square test. The value of p <0.05 was considered statistically significant. Next, to determine the strength of association of the factors of the "Three Delays" with the outcome (fetal death), multiple logistic regression was performed, calculating the crude and adjusted Odds Ratio (OR) with their respective 95% confidence intervals (CI). The Statistical Package for the Social Science (SPSS) version 24 software and R 3·31 software were used to proceed the data analysis. The research was approved by the Ethics Committee in Research / National Commission of Ethics in Research, of the Assis Chateaubriand Teaching Maternity Hospital with the approval n˚ 2,144,962.

## Results

In 2017 there were 5,038 births, of which, 4,929 were born alive and 109 stillborn; yielding a rate of 21.6 stillbirths per 1,000 births. Of the cases analyzed, 86.1% occurred in the antepartum period (before labor) and 13.9% in the intrapartum period (during labor and delivery). Regarding gestational age, 25% of fetal deaths occurred before the 28^th^ week of gestation and 75% occurred after the 28^th^ week of pregnancy, considered as "late fetal death".

The socio demographic and obstetric characteristics of the cases of fetal death and live births are presented in Table 1. As regards prenatal care, it was observed that 12.5% ​​of the women who had stillbirths did not have prenatal consultations, compared to only 1.4% among women who had live births (p = 0.003). Among the stillbirth mothers, 41.7% reported that the gestation was classified as high risk and only 27.7% had prenatal consultations in specialized care services. Regarding childbirth care factors, less than half of the women who had fetal deaths (45%) received guidance on the place where the delivery should be performed and 56.9% of these women had to go to more than one maternity hospital before receiving assistance. Among women with fetal deaths, 25.4% had a cesarean delivery compared to 65.7% of women who had live births (P<0.001). The main maternal complications associated with a cesarean indication were hemorrhagic syndromes in intrapartum deaths and syphilis in antepartum deaths.

**Table 1:** Sociodemographic and obstetric characteristics of cases of fetal deaths and live births. State of Ceará, Brazil, 2017.

Characteristics	Fetal Deaths		Live Births		*P* Value
	n	%	n	%	
**Sociodemographic characteristics**					
**Age group**					0.729
10–14	1	1,4	3	2.1	
15–19	14	19.4	22	15.3	
20–34	47	65.3	92	63.9	
35 or more	10	13.9	27	18.8	
**Education**					0.794
7 years	29	40.3	55	38.2	
8 to 10 years	36	50,0	78	54.2	
11 to 14 years	7	9.7	11	7.6	
**Marital status**					0.145
With partner	54	75.0	120	83.3	
Without partner	18	25,0	24	16.7	
**Characteristics of the mothers**					
**Previous parity**					0.185
Nulliparous	35	48.6	62	43.4	
First live birth	18	25,0	38	26.6	
Multiparous	19	26.4	43	30.1	
**Stillbirth**					0,686
Yes	5	6.9	8	5.6	
No	67	93.1	136	94.4	
**Previous miscarriage**					0.260
Yes	14	19.4	38	26.4	
No	58	80.6	106	73.6	
**Number of prenatal consultations**					**0.003**
None	9	12.5	2	1,4	
1 to 6	42	58.3	79	54.9	
7 or more	20	27.8	58	40.3	
**High-risk pregnancy**					**0.048**
Yes	30	41.7	80	55.9	
No	42	58.3	63	44.1	
**Delivery**					**< 0.001**
C-section	18	25.4	94	65.7	
Vaginal	53	74.6	49	34.3	

Table 2 presents the analysis of factors associated with the "Three Delays" in obstetric care. The analysis found a higher frequency of the three types of delays reported by women who had fetal losses compared to those who had live children.

**Table 2:** The "Three Delays" in obstetric care among cases and controls (fetal deaths and live births). State of Ceará, Brazil, 2017.

Type of Delay	Fetal Deaths		Live Births		*P* Value
	n	%	n	%	
**1. Delay in recognizing the need for care (Family / Patient)**					
**Delay in seeking care**					**< 0.001**
Yes	33	45.8	6	4.2	
No	39	54.2	138	95.8	
**Did not know the signs of risk**					**< 0.001**
Yes	27	37.5	3	2.1	
No	45	62.5	141	97.9	
**Refusal of Care**					**< 0.001**
Yes	16	22.2	5	3.5	
No	56	77.8	139	96.5	
**2. Delay in access to care**					
**Absence or inadequacy of prenatal care**					**< 0.001**
Yes	59	81.9	52	36.1	
No	13	18.1	92	63.9	
**Difficulty with transportation**					0.845
Yes	5	6.9	9	6.3	
No	67	93.1	135	93.8	
**Geographical Difficulty**					0.315
Yes	29	40.3	48	33.3	
No	43	59.7	96	66.7	
**3. Delay in receiving quality care in the Unit**					
**Delay in Diagnosis**					**< 0.001**
Yes	37	51.4	5	3.5	
No	35	48.6	139	96.5	
**Regulatory Difficulty**					**0.019**
Yes	28	38.9	34	23.6	
No	44	61.1	110	76,4	
**Lack of Trained Personnel**					**< 0.001**
Yes	17	23.6	9	6.3	
No	55	76,4	135	93.8	
**Delay in Case Transfer**					**< 0.001**
Yes	33	45.8	23	16.0	
No	39	54.2	121	84.0	
**Delayed onset of treatment**					**< 0.001**
Yes	52	72.2	51	35.4	
No	20	27.8	93	64.6	
**Inadequate conduct with the patient**					**< 0.001**
Yes	28	38.9	8	5.6	
No	44	61.1	136	94.4	

The delays with the significant differences between cases and controls were: absence or inadequacy of prenatal care (81.9% in the cases and 36.1% in the controls), delayed treatment initiation (72.2% vs 35.4% %) and delayed diagnosis (51.4% vs 3.5%). All factors associated with the first "delay in seeking care" and the third delay “receiving adequate care in the unit” was strongly associated with the fetal death outcome. In relation to the second delay, only absence or inadequacy of prenatal care was associated.

The multivariate logistic regression presented in Table 3 was performed to determine the strength of the association between risk factors of the "Three Delays" and the occurrence of fetal deaths. After adjusted analysis, the variables that persisted significantly associated with fetal death were: refusal of care (OR = 4.07, 95% CI: 1.08–15.3), absence or inadequacy of prenatal care (OR = 2.69; 95% CI: 1.07–6.75), delay in diagnosis (OR = 10.3, 95% CI: 2.58–41.4), and inadequate conduct with the patient (OR = 4.88, 95% CI: 1.43–16.6) (Table 3).

**Table 3:** Logistic regression model for the delays in obstetric care associated with the outcome of fetal death. State of Ceará, Brazil, 2017.

Measures "Delays in Obstetric Care"	Crude OR	CI 95%	*P* Value	Adjusted OR	CI 95%	*P* Value
**Delay 1: Delay in recognizing the need for care**						
Delay in seeking care	19.5	7,60–49,8	<0.001	3.42	0.76–15.3	0.108
Did not know the signs of risk	28.2	8.16–97.3	<0.001	3.21	0.50–20.4	0,216
Refusal of Care	7.94	2.77–22.7	<0.001	4.07	1.08–15.3	**0.038**
**Delay 2: Access to the appropriate health facility**						
Absence or inadequacy of prenatal care	8.03	4.02–16.0	<0.001	2.69	1.07–6.75	**0.034**
**Delay 3: Delays in receiving quality care in the Unit**						
Delay in diagnosis	29.3	10.7–80.2	<0.001	10.3	2.58–41.4	**0.001**
Difficulty of regulation	2.05	1.11–3.8	0.020	1.13	0.39–3.26	0.820
Lack of trained staff	4.63	1.94–11.0	0.001	0.64	0.10–3.82	0.628
Delay in case transfer	4.45	2.34–8.47	<0.001	0.55	0.15–1.92	0.348
Delayed onset of treatment	4.74	2.55–8.80	<0.001	1.43	0.48–4.25	0.520
Inadequate conduct with the patient	10.8	4,59–25,4	<0.001	4,88	1.43–16.6	**0.011**

## Discussion

The present study indicates that despite having a higher prevalence of obstetric complications women with fetal death are more likely to refuse obstetric care and are subject to further delays in obstetric care, especially delays in diagnosis and inadequate conducts for their health problem.

These results support the current effort of the World Health Organization and the International Stillbirth Alliance (ISA) to promote evidence-based strategies with the objective of preventing fetal death. The study is also aligned to the guide Making Every Baby Count: Audit and Review of Stillbirths and Neonatal Deaths which recommends the use of the "Three Delays" Model to obtain an in-depth analysis of the determinants and modifiable factors in obstetric care. The study looked at delays in three phases defined from the criteria standardized by the World Health Organization (WHO): phase one, related to women and their relatives, refers to the delay in the decision to seek care; stage two, to the difficulty in gaining access to appropriate care; and phase three, related to the delay in receiving adequate care at the health facility, which may result in adverse maternal and fetal outcomes [8].

Although the study did not find a significant association between fetal deaths and sociodemographic factors, the results reflect the differentiated access to health care that still exists among the less favored population. Delays in seeking care are influenced by socioeconomic factors and the organization of the health care network, in contrast to public policy objectives to promote the systemic integration of health actions and services with the provision of continuous, integral and quality care [9].

Studies confirm that in all countries the risk of stillbirth is higher among marginalized populations; access to health services is lower and quality is affected by social inequalities [10–11]. An approach based on the right to universal health care must include the poorest women and their families.

The refusal of care by women and families is an important risk factor included in the first delay group that remained statistically associated with the occurrence of fetal death. This delay was demonstrated by the refusal to attend prenatal appointments being justified by "unknown gestation", "unwanted pregnancy" and "lack of interest". It was noted that a significant proportion of these women presented an overlapping of risk factors and social vulnerability as: unstable marital relationships, used alcohol and/or smoked during pregnancy. A study conducted in Tanzania suggests that the first delay usually occurs with women with unwanted pregnancies and their prevention could have prevented perinatal damage.^7^ Similar results were observed in another Brazilian study that reinforces the need for services with mechanisms to identify these women, who would greatly benefit from early and adequate follow-up [12].

In this study, some women in the presence of risk signs adopted passive behavior, considering the complications as a "normal" process of pregnancy, resulting in worsening of the situation and consequently in fetal death. It is apparent that the current culture determines that pregnant women only seek care when they are in an advanced stage of labor or in emergency situations, thus reflecting, in addition to a strong determinant of fetal death, the failure of the healthcare network in the essential sense of guaranteeing access, connection and accountability, thus increasing the risks of negative outcomes [13].^.^

The analysis evidenced the absence of prenatal appointments as a determinant factor associated with the occurrence of fetal death, confirmed in other studies as a potentially modifiable factor, with interventions towards increasing access and availability of these services that surely would attenuate the stillbirth rates [14–15]. It also indicates the fragility with the principle of universality of access, guaranteed by Brazilian legislation that advocates that basic services, such prenatal care, should cover the entire target population [16].

In order of importance, the delay in the early diagnosis of morbidities and the detection of risk during prenatal appointments was strongly associated with the occurrence of stillbirths, which reflected in the delay to timely treatment to avoid the outcome. A large proportion of women who had fetal loss had complications such as hemorrhage, severe pre eclampsia, urinary tract infection (UTI), syphilis, and diabetes, which may not have been diagnosed in a timely manner to provide adequate treatment. One guideline advocated by Brazilian public policies is that at the start of prenatal care, the pregnant woman must have tests and receive the results in a timely manner, which will support the risk assessment and the specific therapeutic plan for the case [16].

In this present study, a significant association between fetal death and care in other maternity units was observed in the 24 hours prior to the admission of the pregnant woman. It is worth noting the proportion of women (57%) who sought care in other maternity hospitals, surpassing the proportion registered in the “Born in Brazil” Survey [12, 17–18]. Even when the pregnant women seek access to the indicated hospitals they were not promptly attended to. Many of these patients were in preterm labor and/or obstetric complications. This factor was also evidenced by other studies that emphasize that the complication becomes more serious if emergency obstetric care is not accessible [7, 19].

The problem of inequality in the distribution of obstetric beds is reinforced by the greater concentration of health services in large urban centers, with cities in the interior lacking qualified care, thus increasing demand on maternity hospitals in the capital [20]. These delays in care were also found in other studies that established that the expectation of hospitalization not being met, in addition to identifying inadequacies in care, may represent a potential risk to maternal and fetal life. This is configured as an institutional violence [15].

Another factor observed in the research was the delay in transferring the pregnant woman to appropriate hospital, associated with late diagnosis and incorrect action by the health team. All these deficiencies in the quality of health care delay and limit individuals’ access to procedures that could prevent death [3]. Another study reinforces that one reason for the high rates of stillbirths in low- and middle-income countries is the delay that many women experience in receiving adequate care, including delays in recognizing high-risk maternal disorders, providing transportation and inadequate care facilities [21]. The misinterpretation of clinical signs and mismanagement are also an important contributing factor for intrapartum deaths [7]. Difficulty in accessing health services at different levels, as well as inadequate care, reinforces pregnant women’s lack of trust and affects their decision to seek care until the severity of their condition overcomes all barriers. On the other hand, professionals are also affected by the disorganization of the health system and delay the decision to refer the pregnant women to the appropriate care in each case.

The high percentage of cases of fetal deaths related to vaginal deliveries (74.6%) was apparent in the present study. This result corresponds to the recommendation of the Brazilian Federation of Associations of Gynecology and Obstetrics and other global organizations, which indicate the route of vaginal delivery as preferential for pregnant women with fetal death [22]. According to the literature, fetal death is not indicative of cesarean section and should be reserved for specific conditions, such as complete-total placenta previa, repeated cesarean sections placenta detachment [23].

The frequency of cesarean deliveries has increased in high-income countries and in many middle-income countries, in part because of growing concern about the risk of fetal death. Many studies point to cesarean as a protective factor and recommend access to cesarean sections to reduce intrapartum fetal death. However, the reduction in fetal mortality is more related to the quality of obstetric care than to the way of delivery [7,21,24–25]. When the death has already been confirmed, the best mode of delivery is usually through the vaginal route due to the lower rates of complications in this procedure [10]. Many adverse events or incidents may have their origin in the inadequate application or even the non-application of evidence-based best practices to deal with specific situations. Therefore, the development and implementation of clinical guidelines and optimized protocols should be part of the actions of a program to promote quality in maternal and perinatal care [16].

It was considered as one of the limitations of this study the possibility of selection bias, as it was carried out in a reference maternity hospital for high-risk population with high rates of obstetric and fetal complications. However it was possible to obtain information on all levels of health care and acquire a sample size calculated to verify the differences between groups. The possibility of inadequate registration of miscarriages classified as fetal death was verified by the research team and duly corrected.

It is important to emphasize the importance of this study to reaffirm fetal mortality as a sensitive marker of the quality of care provided to pregnancy and delivery, the health system's resolution and a direct measure of access and quality of intrapartum care [4]. We recognize the recent advances in Brazil related to the improvement of maternal and child indicators, however, the challenge that remains is to transform recommendations into practices, with the services adopting protocols based on scientific evidence, with treatment that is dignified and respectful to women. Improvements are necessary, especially in the sense of promoting the safety and protection of pregnant women throughout the puerperal gestation cycle, thereby regaining the health services’ credibility [7,9,15,17].

The prevention of stillbirth can't be an individualized theme, strategies to reduce these deaths require coordinated and continuous actions at the various levels of attention. It is proven that family-centered approaches and women supported by health care providers can encourage continuity of care. These are some improvements that should be pushed forward with substantial results for both maternal health and the reduction of preventable fetal deaths, thereby reducing the impact of the "three delays".
